# *In situ* embryo rescue for generation of wide intra‐ and interspecific hybrids of *Panicum virgatum* L.

**DOI:** 10.1111/pbi.12573

**Published:** 2016-06-01

**Authors:** Albert P. Kausch, Michael Tilelli, Joel Hague, Christopher Heffelfinger, David Cunha, Maria Moreno, Stephen L. Dellaporta, Kimberly Nelson

**Affiliations:** ^1^ Department of Cell and Molecular BiologyUniversity of Rhode IslandKingstonRIUSA; ^2^ Department of Molecular, Cellular and Developmental BiologyYale UniversityNew HavenCTUSA

**Keywords:** wide crosses, switchgrass, tissue culture, transgenic plants, genetic transformation

## Abstract

Wide crosses have been used for decades as a method for transferring novel genetic material and traits in plant breeding. Historically, many products of wide crosses require tedious and inefficient surgical embryo rescue prior to embryo abortion to recover single plantlets. We have utilized transgenic switchgrass (*Panicum virgatum* L. cv Alamo) as a pollen donor in conjunction with antibiotic or herbicide selection for recovery of intra‐and interspecific F_1_ crosses by using developing ovules from the female parent and selecting for embryogenic cultures derived from the *in situ* immature embryo. Using this approach, several intravarietial crosses were generated between transgenic Alamo and the switchgrass varieties Kanlow, Blackwell and Cave**‐**in**‐**Rock as well as an interspecific cross with Atlantic coastal panicgrass. This procedure selected F_1_ embryogenic callus produced from the developing embryo contained within isolated immature ovules. Several clonal plants were successfully regenerated from each cross. Southern blot, PCR, phenotypic analyses and genomic analysis confirmed F_1_ hybrids. Using genotyping‐by‐sequencing shows the hybridization of the recovered plants by determining the ratio of transgressive markers to total compared markers between parents and their potential offspring. The ratio of transgressive markers to total compared markers was significantly lower between parents and their predicted offspring than between parents and offspring unrelated to them. This approach provides the possibility to move useful transgenes into varieties that are recalcitrant to direct transformation which can be optionally segregated thus useful to create new hybrids, as well as recovery of wide crosses that are either difficult or impossible using traditional techniques.

## Introduction

Switchgrass (*Panicum virgatum* L.) is a semi‐domesticated plant which is highly heterozygous and an anemophilous obligate outcrosser with both prefertilization and postfertilization incompatibility systems present (Martinez‐Reyna and Vogel, 1998, 2002). Two main ecotypes (‘lowland’ and ‘upland’ types) have been characterized and described by geographical adaptation (Casler *et al*., 2011). These two ecotypes show distinct morphological and physiological characteristics (Casler *et al*., 2011; Ersoz *et al*., 2012; Narasimhamoorthy *et al*., 2008), and the genetic distinctiveness of their nuclear genomes has been established (Eberhardt and Newell, 1959; Hopkins *et al*., 1996; McMillan and Weiler, 1959). Ploidy levels vary within switchgrass ecotypes (Eberhardt and Newell, 1959; Hopkins *et al*., 1996; McMillan and Weiler, 1959), with lowland ecotypes identified as mainly as psuedotetraploids or allotetraploids (2*n *= 4x = 36, with 3.1 pg DNA per nucleus), while upland types can be tetraploids or octaploids (2*n *= 8x = 72, with 5.2 pg DNA per nucleus) (Costich *et al*., 2010; Nielsen, 1944).

One problem is a limited ability to introgress and combine useful traits in switchgrass into regionally selected germplasm (such as lowland and upland switchgrass varieties and other species) and some ecotypes are recalcitrant to transformation. However, with ample phenotypic and genetic diversity characterized within and among switchgrass cultivars and populations, data on hybrid plant development are somewhat limited by the time‐consuming and laborious process to recover hybrid plants and fertile alloploids (Hultquist *et al*., 1996; Martinez‐Reyna and Vogel, 2002).

Intervarietal, interspecific and intergeneric, or more distantly related crosses collectively are referred to as ‘wide crosses’. Breeding of wide crosses is most often prevented through either pre‐ or postfertilization incompatibility mechanisms. For example, Martinez‐Reyna and Vogel (Martinez‐Reyna and Vogel, 2002) demonstrated a prefertilization system similar to the S‐Z incompatibility system previously characterized in the Poaceae family (Hayman, 1956; Lundqvist, 1965) exists in *Panicum* sp. (Martinez‐Reyna and Vogel, 2002). Postfertilization incompatibility was indicated as the main obstacle to successful seed development in many switchgrass hybridizations (Martinez‐Reyna and Vogel, 2002) with seed abortion typically occurring between 10 and 30 days post pollination. However, these would be ideal candidates for wide cross embryo rescue hybrids.

Controlled hybridization techniques, based on floral emasculation and mutual pollination by bagging inflorescences, have been used in recovering both population hybrids and specific hybrids of switchgrass (Hultquist *et al*., 1996; Martinez‐Reyna and Vogel, 2002, 2008), and Zhang *et al*. (2011) have demonstrated that these also occur in open pollination. Through these techniques, intraspecific crosses between upland and lowland ecotypes and between spatially separated populations have yielded viable hybrid plants (Hultquist *et al*., 1996; Martinez‐Reyna and Vogel, 1998, 2002, 2008). Although these methods are accurate and promising, they are tedious, time‐consuming and produce low numbers of candidate breeding progeny. Additionally, analysis and verification of hybrid plants require extensive phenotypic observation and measurements based on morphological characteristics before molecular analysis can verify the hybrid genotype. An improved ability to recover wide crosses would be promising.

Endosperm degeneration has been identified as the primary mechanism behind the failure of interspecific and intraspecific interploid crosses in many plants which exhibit postfertilization incompatibility (Brink and Cooper, 1947). In wide crosses which are not prevented by prefertilization incompatibility, the technique of embryo rescue overcomes seed abortion that occurs through abnormal endosperm development by surgically excising the immature embryo and germinating or culturing it on artificial media, independent of the endosperm.

Embryo rescue uses manual surgical recovery of an immature embryo arising usually from an interploid hybrid cross and culturing the embryo *in vitro* (Monnier, 1990) and subsequently culturing the embryo to a whole plant (fertile or infertile). Typically, the postexcision embryo is germinated directly on an appropriate medium. In some species, it may not be technically feasible to surgically excise embryos out of fertilized ovules. In addition, effects of the maternal tissue (especially the ovular wall) may be deleterious to embryo rescue, further contributing to low yields.

The outcome of both surgical removal embryo rescue and immature ovule or caryposis culture techniques is usually a single plantlet. While wide crosses have proven valuable in breeding hybrids, the method of conventional embryo rescue is burdened for a variety of reasons, which limit its application to certain plants and breeding schemes.

Genetic modification will be applied to biofuel crop development (Kausch *et al*., 2010a,b,c; Yuan *et al*., 2008), and switchgrass has been routinely genetically modified (Burris *et al*., 2009; Li and Qu, 2011; Liu *et al*., 2015; Richards *et al*., 2001; Somleva, 2006; Somleva *et al*., 2002; Xi *et al*., 2009) with reliance on cv Alamo. *Agrobacterium*‐mediated transformation has been applied to switchgrass cvs Alamo and Kanlow with generally low numbers of T‐DNA insertions and stable transmission to progeny as Mendelian loci without rearrangements (Liu *et al*., 2015; Somleva *et al*., 2002; Xi *et al*., 2009). Other cultivars including upland varieties cvs Dacotah and Blackwell have recently been transformed (Liu *et al*., 2015), yet other cultivars remain recalcitrant.

We have hypothesized that through the application of a transgenic selectable marker, such as herbicide or antibiotic resistance contributed through the male parent, it would be possible to selectively culture a fertilized embryo developed from intravarietal or interspecific crosses into embryogenic callus from the *in situ* immature embryos in the ovule. The embryogenic callus could then be proliferated into multiple clonal events and regenerated under selection to produce hybrid individual plantlets.

To test this hypothesis, we used transgenic Alamo switchgrass lines as paternal parents in intervarietal and interspecific wide crosses and selected for transgenic T_1_ hybrid embryogenic callus and hybrid plant regeneration.

## Results

### Generation and analysis of transgenic T_0_ lines

**Figure 1:**
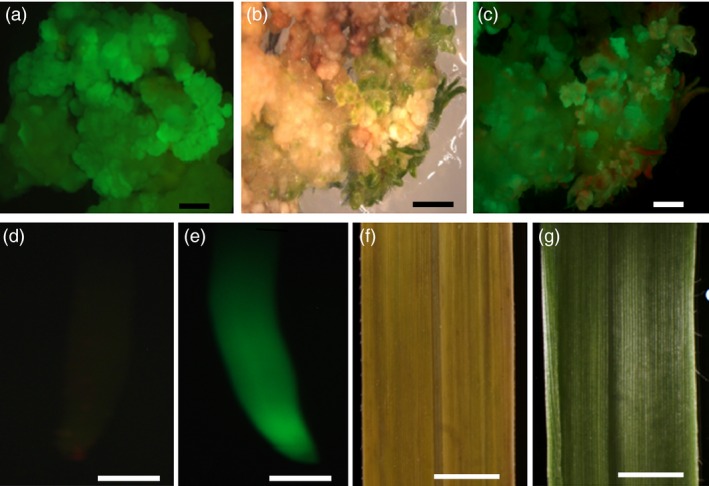
Generation and analysis of transgenic T_0_ lines. (a) GFP image of callus from p35S**‐** *hph:Ubi* ^*R*^ *‐gfp*. (b) Brightfield image of regenerating callus from p35S**‐** *hph:Ubi* ^*R*^ *‐gfp*. (c) Corresponding GFP image to (b). Scale bars 2 mm (a–c). (d) GFP image of wild‐type nontransformed cv Alamo root, scale bar 0.75 mm (e) GFP image of p35S**‐** *hph:Ubi* ^*R*^ *‐gfp* T_0_transformed cv Alamo root scale bar, 0.75 mm (f) Finale treated wild‐type nontransformed cv Alamo leaf, scale bar 5 mm (g) Finale treated transformed p35S**‐** *bar* cv Alamo leaf, scale bar 5 mm.

Selection of resistant embryogenic colonies occurred in *Panicum virgatum* cv Alamo over a 6‐ to 8‐week period under either hygromycin and bialaphos selection. Several transgenic events were generated using both hygromycin and bialaphos selection. Callus from p35S**‐** *hph:Ubi* ^*R*^ *‐gfp* transformants showed GFP expression (Figure 1a) as did their regenerating plants (Figure 1b and c) which were grown to maturity in the greenhouse. Roots from mature plants of WT Alamo are GFP negative (Figure 1d), whereas roots from p35S**‐** *hph:Ubi* ^*R*^ *‐gfp* T_0_ were GFP positive (Figure 1e). Leaves of WT Alamo swabbed with 3% (v/v) Finale showed sensitivity (Figure 1f), whereas those of the T_0_ p35S‐*bar* transgenic regenerated plants scored for resistance to the herbicide (Figure 1g). PCR and Southern blotting were performed to determine the presence, number and structure of T‐DNA insertion(s) carried by the p35S**‐** *hph:Ubi* ^*R*^ *‐gfp* and p35S‐*bar* events (data not shown). Wild‐type cvs Alamo, Kanlow, Blackwell, Cave‐in‐Rock and Atlantic coastal panicgrass (*Panicum aramrum* Ell. var. *amarulum*; hereafter referred to as ACP) were negative for the presence of the transgenes in both assays. All T_0_ switchgrass plants were grown in soil in 10‐inch pots and flowered in the greenhouse. The T_0_ plants were morphologically normal with respect to leaf, root, shoot and flower development and fertility in comparison with wild‐type nontransgenic plants.

### *In situ* recovery of embryogenic callus and hybrid plants

**Figure 2:**
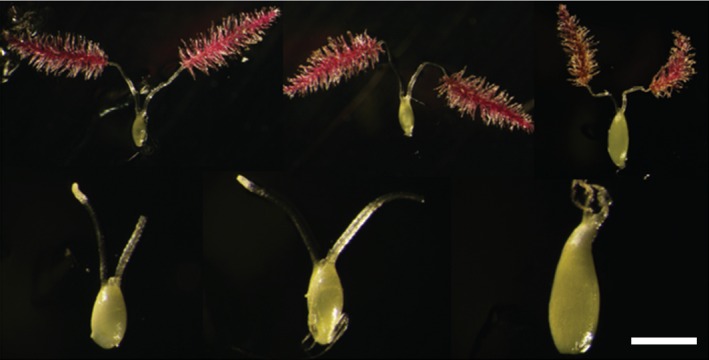
Composite of representative stages of maternal ovules explanted for *in situ* embryo rescue from *P. virgatum* cv Alamo; Scale bar 5 mm.

Independent transgenic events determined to carry single T‐DNA insertions were chosen for hybrid crosses wherein T_0_ plants were backcrossed to wild‐type parents (cvs Alamo, Kanlow, Blackwell, Cave‐in‐Rock and ACP) in pollen cages in growth chambers. Maturing surface‐sterilized ovules from crosses were plated onto callus induction media for 14 days before being placed onto media containing either hygromycin or bialaphos. No attempt was made to optimize the stage of ovule development, and various stages were plated from numerous experiments (Figure 2). Initially, as controls, crosses were set up using an Alamo transgenic plant (p35S‐*hph:Ubi* ^*R*^ *‐gfp* or p35S‐*bar*) and either an Alamo or Kanlow wild‐type plant to demonstrate that this procedure would successfully produce F_1_ plants (Table 1). Both of these crosses resulted in the recovery of resistant embyrogenic calli and plants. From pooled experiments using wild‐type Alamo as the maternal parent, 223 explanted ovules generated 198 initial calli, and these initial calli generated 27 hygromycin‐resistant calli. Similarly, for the *p35S‐bar* experiments using an Alamo wild‐type maternal plant, 267 ovules generated 124 initial calli which resulted in 12 bialaphos‐resistant calli colonies. Using a wild‐type Kanlow plant as the maternal parent in the p35S‐*hph:Ubi* ^*R*^ *‐gfp* cross, 35 explanted ovules generated 20 initial calli of which 5 were hygromycin‐resistant. No bialaphos‐resistant Kanlow plants were recovered from 28 explanted ovules.

**Table 1:** Pooled experiments with developing ovules from crosses between paternal transgenic plants and wild‐type maternal plants

Maternal WT parent	Paternal transgenic parent	Vector	Ovules plated	Resistant calli/total initial calli	% Plantlet regeneration/resistant calli
*Pancicum virgatum* L. cv Alamo	*Pancicum virgatum* L. cv Alamo	p35S‐*bar*	267	12/124	100%
*Pancicum virgatum* L. cv Alamo	*Pancicum virgatum* L. cv Alamo	p35S‐*hph:Ubi* ^*R*^ *‐gfp*	223	27/198	64.7%
*Pancicum virgatum* L. cv Kanlow	*Pancicum virgatum* L. cv Alamo	p35S‐*hph:Ubi* ^*R*^ *‐gfp*	35	5/20	40.0%
*Pancicum virgatum* L. cv Blackwell	*Pancicum virgatum* L. cv Alamo	p35S‐*bar*	113	14/34	50.0%
*Pancicum virgatum* L. cv Blackwell	*Pancicum virgatum* L. cv Alamo	p35S‐*hph:Ubi* ^*R*^ *‐gfp*	65	5/33	66.7%
*Pancicum virgatum* L. cv Cave‐in‐Rock	*Pancicum virgatum* L. cv Alamo	p35S‐*hph:Ubi* ^*R*^ *‐gfp*	77	3/27	50.0%
*Pancicum aramum* L. cv Amularum	*Pancicum virgatum* L. cv Alamo	p35S‐*hph:Ubi* ^*R*^ *‐gfp*	202	10/139	30.0%

**Figure 3:**
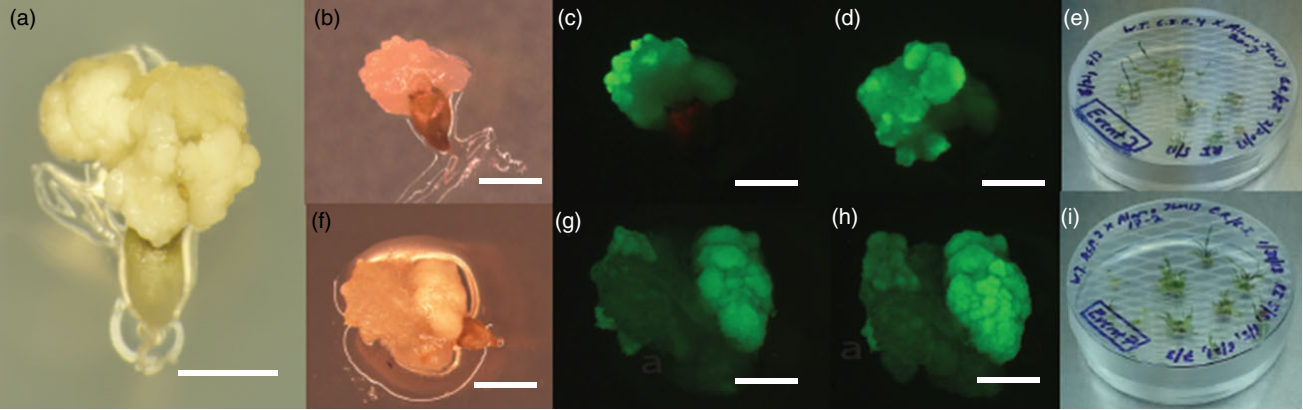
*In situ* embryo rescue from maternal ovules derived from intervarietal and interspecific crosses. (a) initial embryogenic callus derived from a representative explanted ovule three weeks after culture on medium without selection. Scale bar equals 5 mm (b) brightfield image of *P. virgatum* cv Cave‐in‐Rock × cv Alamo p35S**‐** *hph:Ubi* ^*R*^ *‐gfp* ovule on hyg selection (c) same ovule as (b) imaged for GFP (d) same ovule as (c) imaged for GFP after five days growth (e) clonal plantlets derived from a single embryo rescue using p35S**‐** *hph:Ubi* ^*R*^ *‐gfp* (f) Brightfield image of *P. amarum* cv Amularum × cv Alamo p35S**‐** *hph:Ubi* ^*R*^ *‐gfp* ovule on hyg selection (g) same ovule as (f) imaged for GFP (h) same ovule as (g) imaged for GFP after five days growth (i) clonal plantlets derived from a single embryo rescue using p35S**‐** *hph:Ubi* ^*R*^ *‐gfp*, scale bar for (b–h) is 2.5 mm.

Subsequently, crosses were established using an Alamo transgenic plant (p35S‐*hph:Ubi* ^*R*^ *‐gfp* or p35S‐*bar*) and either a wild‐type Blackwell, Cave‐in‐Rock or ACP plant. Embryogenic calli generated from a representative ovule of these crosses is shown in Figure 3. In the crosses using the hygromycin‐resistant, GFP‐positive Alamo plant, GFP‐positive F_1_ colonies from plated ovules were observed between two and fourteen days post plating. For the cross using a wild‐type Blackwell plant as the maternal parent and a p35S‐*hph:Ubi* ^*R*^ *‐gfp*‐positive Alamo plant as the parental parent, 65 initial ovules were plated, resulting in 33 initial calli and 5 resistant calli. For the crosses using a wild‐type Blackwell plant as the maternal parent and a p35S‐*bar* Alamo parent as the paternal parent, 113 ovules were excised, with 34 initial calli formed and 14 bialaphos‐resistant calli formed. Using Cave‐in‐Rock as the maternal parent and p35S‐*hph:Ubi* ^*R*^ *‐gfp* Alamo pollen donor, ten hygromycin‐resistant calli were recovered from 139 initial calli. In an interspecific cross using wild‐type ACP as the maternal parent and crossed to p35S‐*hph:Ubi* ^*R*^ *‐gfp* Alamo plant resulted in ten events from 139 initial calli derived from 202 plated ovules. All hygromycin‐resistant colonies were GFP positive (Figure 3).

### Phenotypic and molecular characterization of T_1_ hybrids

**Figure 4:**
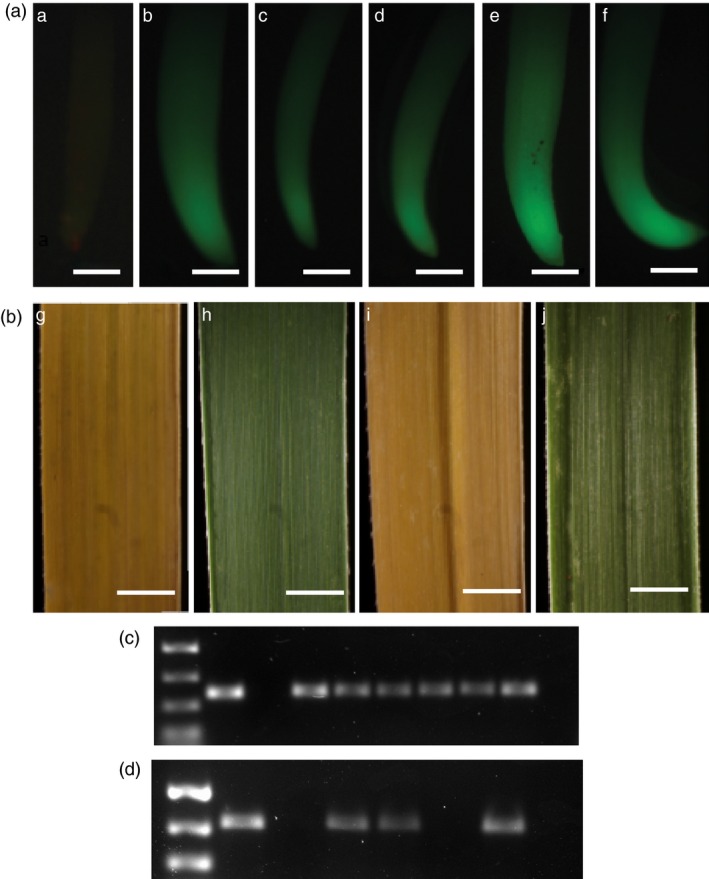
Analysis of GFP and BAR expression in T_1_ embryo rescue derived plants. (A) GFP imaging of roots from WT cv Alamo, parental p35S**‐** *hph:Ubi* ^*R*^ *‐gfp* cv Alamo and T_1_ hybrid plants (T_1_H) derived from crosses to p35S**‐** *hph:Ubi* ^*R*^ *‐gfp* cv Alamo parents. (a) WT cv Alamo (b) p35S**‐** *hph:Ubi* ^*R*^ *‐gfp* cv Alamo (c) T_1_H Alamo × Kanlow (d) T_1_H Alamo × Blackwell (e) T_1_H Alamo × Cave ‐n‐Rock and (f) T_1_H Alamo × ACP. Scale bar (a–f) = 0.75 mm. (B) Paint assay results using 3% Finale on mature leaves of WT plants, parental p35S‐*bar* cv Alamo and T_1_ hybrid plants (T_1_H) derived from crosses with p35S**‐** *bar* cv Alamo parents. (g) WT cv Alamo (h) T_1_H Alamo × Alamo (i) WT cv Blackwell and (j) T_1_H plant Alamo × Blackwell. Scale bar (g–j) = −5 mm (C) PCR amplification of a 364‐bp amplicon within the sGFP cassette of p35S**‐** *hph:Ubi* ^*R*^ *‐gfp*; lane 1, NEBL PCR marker, 755‐bp to 150‐bp bands shown; lane 2, amplification from p35S**‐** *hph:Ubi* ^*R*^ *‐gfp*; lane 3, WT type Alamo, negative control; lanes 4–9 correspond with (b–f) of (A); lane 10 is NTC. (D) PCR amplification of a 513‐bp amplicon within the *bar* cassette of p35S‐*bar*; lane 1, NEBL PCR marker, 755‐bp to 300‐bp band shown; lane 2, amplification from p35S‐*bar*; lane 3, WT Alamo; lanes 4 and 5, two individual T_1_H p35S‐*bar* Alamo × WT Alamo plants; lane 6, WT cv Blackwell; lane 7, T_1_H p35S‐*bar* Alamo × WT Blackwell plant; lane 8 is NTC.

Numerous clones from both the hygromycin‐ and bialaphos‐resistant colonies were successfully regenerated and grown to maturity in the greenhouse (Figures 3e, i). Plant regeneration in tissue culture was normal as compared to transgenic T_0_ plants_._ As expected, control wild‐type Alamo plants are negative for GFP expression in their roots (Figure 4a). Mature plants derived from crosses between wild‐type Alamo, Kanlow, Blackwell, Cave ‐in‐Rock and ACP maternal parents and the p35S‐*hph:Ubi* ^*R*^ *‐gfp* Alamo paternal parent all showed GFP expression in their roots in F_1_ plants (Figure 4b–f). Also, as expected, control wild‐type Alamo and wild‐type Blackwell were sensitive to bialaphos (3% Final v/v) (Figure 4g, i), whereas hybrid plants derived from wild‐type Alamo and wild‐type Blackwell crossed with p35S‐*bar* Alamo tested positive for bialaphos resistance in leaf swab assays (Figure 4h, j).

**Figure 5:**
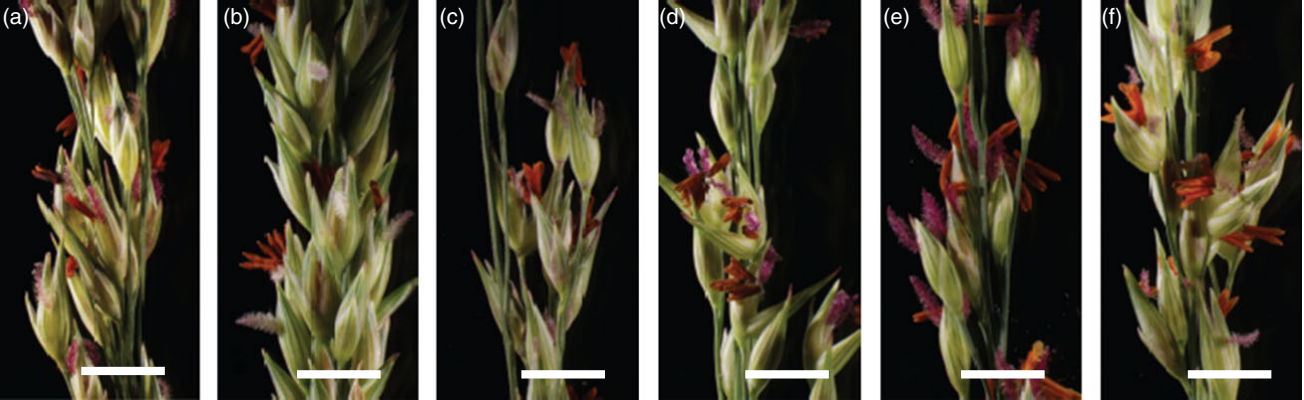
Floral morphology of mature plants of (a) WT cv Alamo and T_1_ mature plants derived from crosses with p35S**‐** *hph:Ubi* ^*R*^ *‐gfp* Alamo and (b) WT Alamo, (c) Kanlow, (d) Blackwell, (e) Cave‐In‐Rock and (f) ACP. All flowers and spikelets appear normal at maturity and shed pollen. Scale bars 5 mm.

PCR amplification of a 364‐bp amplicon within the sGFP cassette of confirms the presence of the transgene p35S**‐** *hph:Ubi* ^*R*^ *‐gfp* in the hybrids in comparison with controls (Figure 4C), and PCR amplification of a 513‐bp amplicon within the *bar* cassette of p35S‐*bar* also confirms the presence of this transgene in the hybrids in comparison with controls (Figure 4D). This corresponds to the phenotypic data described above. At maturity, floral morphology of the hybrids were compared to the wild‐type and transgenic parents and were normal in comparison. All hybrid plants had normal panicle (Figure 5a–f) and spikelet morphologies. Additionally, all hybrid plants produced fertile pollen (data not shown) by iodine potassium iodide (IKI) staining (Johansen, 1940).

The F_1_ seedlings were grown to maturity and verified by Southern blot and PCR analysis as the progeny of the T_0_ crosses. All F_1_ plantlets contained an identical fragment to their T_0_ transgenic parent. No contamination from an outside source of transgenic pollen was observed. These data verified that the T‐DNA was stably integrated into the host plant genome and was inherited through germ‐line cells to F_1_ offspring. Similar experiments and analysis were conducted using the T_0_ p35S‐bar Alamo transgenics crossed with WT Kanlow and WT Blackwell.

### Resolution of hybrids by genomics

**Figure 6:**
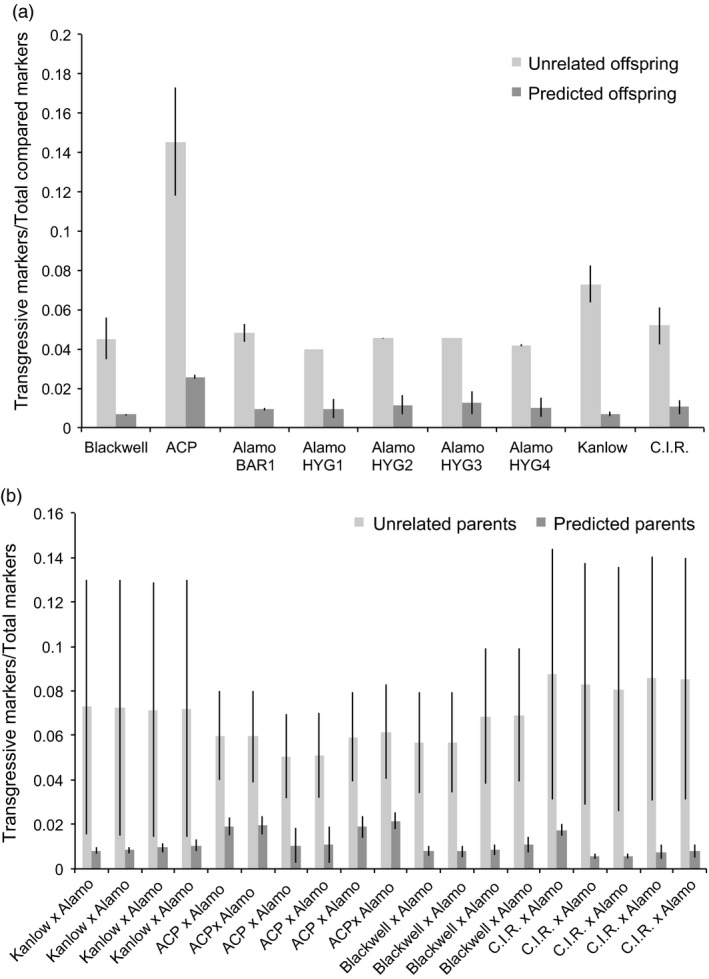
Ratios of transgressive to total compared markers for all parents and offspring. (a) shows comparison between unrelated offspring to predicted offspring using transgressive markers/total compared markers for the various genotypes and trangenic parents. (b) shows comparison between unrelated offspring to predicted offspring using transgressive markers/total markers for the various hybrids. Error bars represent standard deviation of the mean.

To confirm the identity of the parents for each F_1_ offspring, the ratio of sites where neither of the two offspring alleles (transgressive events) were present in the parent was compared to the total number of sites. A transgressive marker is one where neither of the two alleles present in a given offspring were present in a parent. This analysis was performed for every possible parent–offspring combination (Table S1). It was found that every parental variety shared at least one allele per site with its proposed offspring than offspring identified as having different varieties as parents (*P* ≫ 1 × 10^−5^, two‐tailed *t*‐test assuming unequal variances (Figure 6a). For example, between Blackwell and its proposed offspring, only 0.65% (±0.06% (*SD*) of the total markers were transgressive, whereas between Blackwell and unrelated offspring, 4.52% (±1.05% (*SD*) of the compared markers were transgressive. It is worth noting that, as the Alamo p35S‐*hph:Ubi* ^*R*^ *‐gfp* plants were not genetically distinct, they were considered to have the same pool of offspring between plants for this analysis. Ratios of transgressive to total compared markers for all parents and offspring (Figure 6a) show comparison between unrelated offspring to predicted offspring using transgressive markers/total compared markers for the various genotypes and trangenic parents, and in Figure 6b, the comparison between unrelated offspring to predicted offspring using transgressive markers/total markers confirms the various hybrids.

## Discussion

Our results show that postfertilization recovery of hybrid switchgrass plants can be facilitated by the use of transgenic intermediates by using a selectable marker. We have demonstrated that the use of transgenic lines with selectable markers can be used to recover intra‐ and interspecific crosses derived from the developing embryo in the ovule. We show that the transgenes are stably inherited and expressed using selectablable markers or reporter genes. In a previous paper (Heffelfinger *et al*., 2015), we showed that transgenes can be segregated to yield non‐GMO hybrids in F_1_BC_1_ populations. We have also used genomic analysis to confirm the identity of the parents for each F_1_ offspring using the ratio of sites where neither of the two offspring alleles (transgressive events) were present in the parent compared to the total number of sites. These results support that F_1_ individuals show a closer relationship to both of their proposed parents than to nonparental plants. The observed ratios for the resistant calli/total initial calli recovered may be caused by the initial F_1_ callus, which is either segregating for the transgene or is a derivative from maternal tissue. Our results show that the selected callus and its regenerated plants are the result of hybridization.

Here, we also show that useful transgenes can be transferred to varieties (such as Blackwell, Cave‐in‐Rock and ACP) that are recalcitrant to direct transformation and that this technique can be used to create new transgenic hybrids using parents which exhibit postfertilization incompatibility. Immature embryos are often used as an explant source for transformation in monocots and other species. By extension, therefore, it is reasonable that the immature embryo within the developing ovule can be used to select for the products of wide crosses which experience postfertilization incompatibility.

The methods for single embryo rescue are hampered by the tedious techniques and the limitations of their application to certain plants and breeding schemes. Also, this is difficult or impossible to achieve on small seeded plants. Hence, the recovery of hybrids between wide crosses with postfertilization incompatibilities has long been problematic. We are not aware of any studies utilizing transgenic pollen donors and selectable markers for the recovery of wide crosses through the culture of the immature embryo in the ovule. The use of transgenics as ‘bridge intermediates’ to achieve wide cross embryo rescue, where the transgenes can then be removed from fertile hybrids through backcrossing (Heffelfinger *et al*., 2015), is therefore an important breeding tool which technically results in non‐GMO hybrids. This technique for recovery of wide cross‐hybrids may be broadly applicable to many crop species.

## Experimental procedures

### Plant material

Commercially available cultivars of switchgrass (*Panicum virgatum* L.), cvs Alamo, Kanlow, Blackwell, Cave‐in‐Rock and ACP were donated by Ernst Conservation Seeds (Meadville, PA) for use in this study.

### Vectors for genetic transformation

Genetic transformation experiments described herein used two plant transformation vectors designated as: p35S‐*bar* and p35S**‐** *hph:Ubi* ^*R*^ *‐gfp*. The p35S‐*bar* vector *was* constructed in the intermediate vector pSB11 and mobilized into the *Agrobacterium tumefaciens* strain LBA4404 (pSB1) via electroporation as described previously (Hu *et al*., 2008; Komari *et al*., 1996; Luo *et al*., 2004; Luo *et al*., 2006). The binary vector p35S**‐** *hph:Ubi* ^*R*^ *‐gfp* (kindly provided by Dr. Rongda Qu, North Carolina State University) was introduced via electroporation into *Agrobacterium tumefaciens* strain EHA105, and its construction has been described previously as well (Hood *et al*., 1993; Lu *et al*., 2008).

### Generation of T_0_ plants

The primary T_0_ Alamo transgenic plants used in this study were produced using the protocol previously described by Somleva *et al*. (2002). The plants were screened by PCR for the presence of the transgenes. In addition, plants made using the p35S‐*bar* transgene were swabbed with 3% (v/v) Finale (Bayer Environmental Science, Research Triangle Park, NC) to assay for herbicide resistance. Young roots from plants produced using p35S‐*hph:Ubi* ^*R*^ *‐gfp* transgene were visualized for GFP expression using a Zeiss Discovery v20 stereomicroscope with a GFP 470 filter.

### Molecular analyses of transgenic plants

Southern Blot and PCR analyses were carried out essentially as described in Heffelfinger *et al*. (2015). For plants carrying the p35S‐*hph:Ubi* ^*R*^ *‐gfp* transgenes, the GFP transgene was detected via the KAPA 3G Plant PCR kit (KAPA Biosystems, Wilmington, MA) using 5′‐ACGTAAACGGCCACAAGTTC‐3′(forward) and 5′‐TGCTCAGGTAGTGGTTGTCG‐3′(reverse) primers. The manufacturer's directions for crude sample PCR (50 μL reaction) were used in conjunction with the Harris Unicore System. The reaction conditions were 95 °C for 10 min and 30 cycles of 95 °C for 30 s, 55 °C for 15 s and 72 °C for 30 s, followed by 72 °C for 1 min. The resulting PCR products were viewed on a 1.2% agarose gel. The identical primers were employed to prepare a 546‐bp GFP probe using the PCR DIG Probe Synthesis Kit (Roche Applied Science, Indianapolis, IN). This probe was used in Southern analyses of the p35S‐*hph:Ubi* ^*R*^ *‐gfp* transgenics. Transgenics generated using p35S‐*bar* were analysed as described in Heffelfinger *et al*. (2015).

### Hybrid cross set‐up and ovule excision

Just prior to anthesis, switchgrass plants were placed into pollen cages constructed using ½′′ PVC pipe covered with two layers of row cloth (Johnny's Selected Seeds, Winslow, ME). In each pollen cage, two plants were placed for each hybrid testcross: one wild‐type plant and one transgenic Alamo switchgrass plant that was either hygromycin or bar positive by PCR. Plants were allowed to pollinate for 12–25 days with daily agitation and watered daily.

Following the pollination period, the wild‐type individual was removed from the pollen cage and all inflorescences were cut into 1–3′′ sections. The sections were surface sterilized with a 70% ethanol rinse followed by 20% bleach and 0.1% Tween‐20 for 30 min followed by three rinses in sterile distilled water. Fertilized ovules were aseptically dissected out of the florets using a dissection microscope.

### Recovery of embryogenic callus and plant regeneration

Recovered ovules were placed on switchgrass callus induction medium containing 2 mg/L L‐proline and incubated at 25 °C for 2 weeks in the dark. After 2 weeks, callus colonies from individual ovules were plated to switchgrass selection medium containing either 3 mg/L bialaphos or 300 mg/L hygromycin. After 4–6 weeks, resistant embryogenic callus colonies were moved to regeneration I medium and placed into the light (16 h : 8 h) for shoot formation. Small individual plantlets were transferred to plant containers containing regeneration II medium for root development for 2–3 weeks in the light (16 h : 8 h). Individual plants were planted in soil and grown to maturity in the greenhouse.

### Genomics analysis

Genomic DNA was prepared as described in Heffelfinger *et al*. (2015). Twenty‐seven samples (eight parents and nineteen offspring) were sequenced via genotyping‐by‐sequencing (Heffelfinger *et al*., 2015) on one Illumina Hi‐Seq 2500 lane at the Yale Center for genome analysis. A sample from Blackwell switchgrass was sequenced via whole‐genome sequencing on an Illumina 2000 lane. Reads were aligned against the draft *Panicum virgatum* v. 1.1 reference genome (DOE‐JGI, http://www.phytozome.net/panicumvirgatum) (Goodstein *et al*., 2012) (Novocraft, http://www.novocraft.com/products/novoalign/), and variants were called using GATK (McKenna *et al*., 2010; Van der Auwera *et al*., 2013).

A total of 9 425 423 raw variants were identified in the data set. Raw variation was filtered with the following criteria: a call in every sample supported by at least five sequencing reads, a mapping quality score ≥40, a Phred score ≥40, minimum allele coverage (number of times a reference or nonreference allele appears across all samples) ≥2, quality depth ≥2, fisher strand bias score ≤, f and read position rank sum ≥−8. A total of 12 491 variants were retained after filtering.

To confirm the identities of the parents of each cross, the ratio of sites where neither of the two alleles present in the offspring was present in the proposed parent was compared to the total site count. A minimum depth of coverage of ten reads in both the parent and offspring at a given site was required for it to be included. This analysis was performed for every possible parent–offspring combination across the data set.

## Conflict of interest

This work has been submitted as US Provisional patent application, and otherwise, the authors have no conflict of interest.

## Supporting information


**Table S1** Ratio of transgressive markers to total compared markers between parents and offspring.

## References

[pbi12573-bib-0001] Brink, R.A. and Cooper, D.C. (1947) The endosperm in seed development. Bot. Rev. 13, 423–541.

[pbi12573-bib-0002] Burris, J. , Mann, D. , Joyce, B. and Stewart, C. (2009) An improved tissue culture system for embryogenic callus production and plant regeneration in switchgrass (*Panicum virgatum* L.). Bioenergy Res. 2, 267–274.

[pbi12573-bib-0003] Casler, M.D. , Tobias, C.M. , Kaeppler, S.M. , Buell, C.R. , Wang, Z.‐Y. , Cao, P. , Schmutz, J. *et al* (2011) The switchgrass genome: tools and strategies. Plant Genome, 4, 273–282.

[pbi12573-bib-4000] Costich, D.E. , Friebe, B. , Sheehan, M.J. , Casler, M.D. and Buckler, E.S. (2010) Genome‐size variation in switchgrass (*Panicum virgatum*): flow cytometry and cytology reveal rampant aneuploidy. Plant Genome, 3, 130–141.

[pbi12573-bib-1000] Eberhardt, S.A. and Newell, L.C. (1959) Variation in domestic collections of swtichgrass, *Panicum virgatum* . Agron. J. 51, 613–616.

[pbi12573-bib-0004] Ersoz, E.S. , Wright, M.H. , Pangilinan, J.L. , Sheehan, M.J. , Tobias, C. , Casler, M.D. , Buckler, E.S. *et al* (2012) SNP discovery with EST and NextGen sequencing in switchgrass (*Panicum virgatum* L.). PLoS ONE, 7, e44112. 23049744 10.1371/journal.pone.0044112 PMC3458043

[pbi12573-bib-0005] Goodstein, D.M. , Shu, S. , Howson, R. , Neupane, R. , Hayes, R.D. , Fazo, J. , Mitros, T. *et al* (2012) Phytozome: a comparative platform for green plant genomics. Nucleic Acids Res. 40, D1178–D1186. 22110026 10.1093/nar/gkr944 PMC3245001

[pbi12573-bib-5000] Johansen, D. (1940) Plant Microtechnique. London: McGraw Hill.

[pbi12573-bib-0006] Hayman, D.L. (1956) The genetic control of incompatibility in *Phalaris coerulescens* Desf. Aust. J. Biol. Sci. 9, 321–331.

[pbi12573-bib-0007] Heffelfinger, C. , Deresienski, A.P. , Nelson, K.A. , Moreno, M.A. , Hague, J.P. , Dellaporta, S.L. and Kausch, A.P. (2015) Genomic characterization of interspecific hybrids and an admixture population derived from *Panicum amarum* × *P. virgatum* . Plant Genome, 8, 1–12. 10.3835/plantgenome2015.01.0001 33228322

[pbi12573-bib-0008] Hopkins, A.A. , Taliaferro, C.M. , Murphy, C.D. and Christian, D. (1996) Chromosome number and nuclear DNA content of several switchgrass populations. Crop Sci. 36, 1192–1195.

[pbi12573-bib-1001] Hood, E.E. , Gelvin, S.B. , Melchers, L.S. and Hoekema, A. (1993) New *Agrobacterium* helper plasmids for gene transfer to plants. Transgenic Res. 2, 208–218.

[pbi12573-bib-0009] Hultquist, S.J. , Vogel, K.P. , Lee, D.J. , Arumuganathan, K. and Kaeppler, S. (1996) Chloroplast DNA and nuclear DNA content variations among cultivars of switchgrass, *Panicum virgatum* L. Crop Sci. 36, 1049–1052.

[pbi12573-bib-6000] Hu, Q. , Kononowicz‐Hodges, H. , Nelson‐Vasilchik, K. , Viola, D. , Zeng, P. , Liu, H. , Kausch, A.P. , Chandlee, J.M. , Hodges, T.K. and Luo, H. (2008) FLP recombinase‐mediated site‐specific recombination in rice. Plant Biotechnol J. 6, 176–188. 18021190 10.1111/j.1467-7652.2007.00310.x

[pbi12573-bib-0010] Kausch, A.P. , Hague, J. , Oliver, M. , Li, Y. , Daniell, H. , Mascia, P. and Stewart, C.N. (2010a) Genetic modification in dedicated bioenergy crops and strategies for gene confinement In Plant Biotechnology for Sustainable Production of Energy and Co‐products (Mascia P.N., Scheffran J. and Widholm J.M., eds), pp. 299–315. Berlin Heidelberg: Springer.

[pbi12573-bib-0011] Kausch, A.P. , Hague, J. , Oliver, M. , Li, Y. , Daniell, H. , Mascia, P. , Watrud, L.S. *et al* (2010b) Transgenic perennial biofuel feedstocks and strategies for bioconfinement. Biofuels, 1, 163–176.

[pbi12573-bib-0012] Kausch, A.P. , Hague, J. , Oliver, M. , Watrud, L.S. , Mallory‐Smith, C. , Meier, V. and Stewart, C.N. (2010c) Gene flow in genetically engineered perennial grasses: lessons for modification of dedicated bioenergy crops In Plant Biotechnology for Sustainable Production of Energy and Co‐products (Mascia P.N., Scheffran J. and Widholm J.M., eds), pp. 285–297. Berlin Heidelberg: Springer.

[pbi12573-bib-7000] Komari, T. , Hiei, Y. , Saito, Y. , Murai, N. and Kumashiro, T. (1996) Vectors carrying two separate T‐DNAs for co‐transformation of higher plants mediated by *Agrobacterium tumefaciens* and segregation of transformants free from selection markers. Plant J. 10, 165–174. 8758986 10.1046/j.1365-313x.1996.10010165.x

[pbi12573-bib-0013] Li, R. and Qu, R. (2011) High throughput Agrobacterium‐mediated switchgrass transformation. Biomass Bioenergy, 35, 1046–1054.

[pbi12573-bib-0014] Liu, Y.‐R. , Cen, H.‐F. , Yan, J.‐P. , Zhang, Y.‐W. and Zhang, W.‐J. (2015) Inside out: high‐efficiency plant regeneration and Agrobacterium‐mediated transformation of upland and lowland switchgrass cultivars. Plant Cell Rep. 34, 1099–1108. 25698105 10.1007/s00299-015-1769-x

[pbi12573-bib-0777] Lu, J. , Sivamani, E. , Li, X. and Qu, R. (2008) Activity of the 5’ regulatory regions of the rice polyubiquitin rubi3 gene in transgenic rice plants as analyzed by both GUS and GFP reporter genes. Plant Cell Rep. 27, 1587–1600. 18636262 10.1007/s00299-008-0577-y

[pbi12573-bib-0015] Lundqvist, A. (1965) Self‐incompatibility in *Dactylis aschersoniana* Graebn. Hereditas, 54, 70–87.

[pbi12573-bib-8000] Luo, H. , Hu, Q. , Nelson, K. , Longo, C. , Kausch, A.P. , Chandlee, J.M. , Wipff, J.K. and Fricker, C.R. (2004) *Agrobacterium tumefaciens*‐mediated creeping bentgrass (*Agrostis stolonifera* L.) transformation using phosphinothricin selection results in a high frequency of single‐copy transgene integration. Plant Cell Rep. 22, 645–652. 14615907 10.1007/s00299-003-0734-2

[pbi12573-bib-9000] Luo, H. , Lee, J.Y. , Hu, Q. , Nelson‐Vasilchik, K. , Eitas, T.K. , Lickwar, C. , Kausch, A.P. , Chandlee, J.M. and Hodges, T.K. (2006) RTS, a rice anther‐specific gene is required for male fertility and its promoter sequence directs tissue‐specific gene expression in different plant species. Plant Mol Biol. 62, 397–408. 16897470 10.1007/s11103-006-9031-0

[pbi12573-bib-0016] Martinez‐Reyna, J.M. and Vogel, K.P. (1998) Controlled hybridization technique for switchgrass. Crop Sci. 38, 876–878.

[pbi12573-bib-0017] Martinez‐Reyna, J.M. and Vogel, K.P. (2002) Incompatibility systems in switchgrass. Crop Sci. 42, 1800–1805.

[pbi12573-bib-0018] Martinez‐Reyna, J.M. and Vogel, K.P. (2008) Heterosis in switchgrass: spaced plants. Crop Sci. 48, 1312–1320.

[pbi12573-bib-2000] McMillan, C. and Weiler, J. (1959) Cytogeography of *Panicum virgatum* in central North America. Am. J. Bot. 46, 590–593.

[pbi12573-bib-0019] McKenna, A. , Hanna, M. , Banks, E. , Sivachenko, A. , Cibulskis, K. , Kernytsky, A. , Garimella, K. *et al* (2010) The Genome Analysis Toolkit: a MapReduce framework for analyzing next‐generation DNA sequencing data. Genome Res. 20, 1297–1303. 20644199 10.1101/gr.107524.110 PMC2928508

[pbi12573-bib-0020] Monnier, M. (1990) Culture of zygotic embryos of higher plants In Plant Cell and Tissue Culture (Pollard J.W. and Walker J.W., eds), pp. 129–139. Totowa: Humana Press. 10.1385/0-89603-161-6:129 21390601

[pbi12573-bib-0021] Narasimhamoorthy, B. , Saha, M. , Swaller, T. and Bouton, J. (2008) Genetic diversity in switchgrass collections assessed by EST‐SSR markers. Bioenergy Res. 1, 136–146.

[pbi12573-bib-0022] Nielsen, E.L. (1944) Analysis of variation in *Panicum virgatum* . J. Agric. Res. 69, 327–353.

[pbi12573-bib-0023] Richards, H.A. , Rudas, V.A. , Sun, H. , McDaniel, J.K. , Tomaszewski, Z. and Conger, B.V. (2001) Construction of a GFP‐BAR plasmid and its use for switchgrass transformation. Plant Cell Rep. 20, 48–54. 10.1007/s002990000274 30759912

[pbi12573-bib-0024] Somleva, M.N. (2006) Switchgrass In Agrobacterium Protocols (Wang K., ed), pp. 65–74. Totowa, NJ: Humana Press.

[pbi12573-bib-0025] Somleva, M.N. , Tomaszewski, Z. and Conger, B.V. (2002) Agrobacterium‐mediated genetic transformation of switchgrass. Crop Sci. 42, 2080–2087.

[pbi12573-bib-1200] Van der Auwera, G.A. , Carneiro, M.O. , Hartl, C. , Poplin, R. , del Angel, G. , Levy‐Moonshine, A. , Jordan, T. , Shakir, K. , Roazen, D. , Thibault, J. , Banks, E. , Garimella, K.V. , Altshuler, D. , Gabriel, S. and DePristo, M.A. (2013) From FastQ data to high‐confidence variant calls: the Genome Analysis Toolkit best practices pipeline. Curr. Protoc. Bioinformatics, 43, 1–33. 25431634 10.1002/0471250953.bi1110s43 PMC4243306

[pbi12573-bib-0027] Xi, Y. , Fu, C. , Ge, Y. , Nandakumar, R. , Hisano, H. , Bouton, J. and Wang, Z.‐Y. (2009) Agrobacterium‐mediated transformation of switchgrass and inheritance of the transgenes. Bioenergy Res. 2, 275–283.

[pbi12573-bib-0028] Yuan, J.S. , Tiller, K.H. , Al‐Ahmad, H. , Stewart, N.R. and Stewart, C.N. Jr . (2008) Plants to power: bioenergy to fuel the future. Trends Plant Sci. 13, 421–429. 18632303 10.1016/j.tplants.2008.06.001

[pbi12573-bib-0029] Zhang, Y. , Zalapa, J. , Jakubowski, A.R. , Price, D.L. , Acharya, A. , Wei, Y. , Brummer, E.C. *et al* (2011) Natural hybrids and gene flow between upland and lowland switchgrass. Crop Sci. 51, 2626–2641.

