# Supplementary material for: In situ embryo rescue for generation of wide intra‐ and interspecific hybrids of Panicum virgatum L
Source: Plant Biotechnol J. 2016 Jun 1;14(11):2168–75. doi: 10.1111/pbi.12573 (PMC5095774; doi:10.1111/pbi.12573)
Supplement: Supplementary file 1 — Table S1 Ratio of transgressive markers to total compared markers between parents and offspring. [file PBI-14-2168-s001.docx]

**Supporting Information**

Table S1

**Table S1.**  Ratio of transgressive markers to total compared markers between parents and offspring. A transgressive marker analysis was done for every possible parent-offspring combination and it was found that every parental variety shared at least one allele per site.
